# Supplementary figures and images for: Improving performance of polygenic risk scores for hypertension across two ancestry groups
Source: PLoS One. 2026 Jul 24;21(7):e0353311. doi: 10.1371/journal.pone.0353311 (PMC13399276; doi:10.1371/journal.pone.0353311)

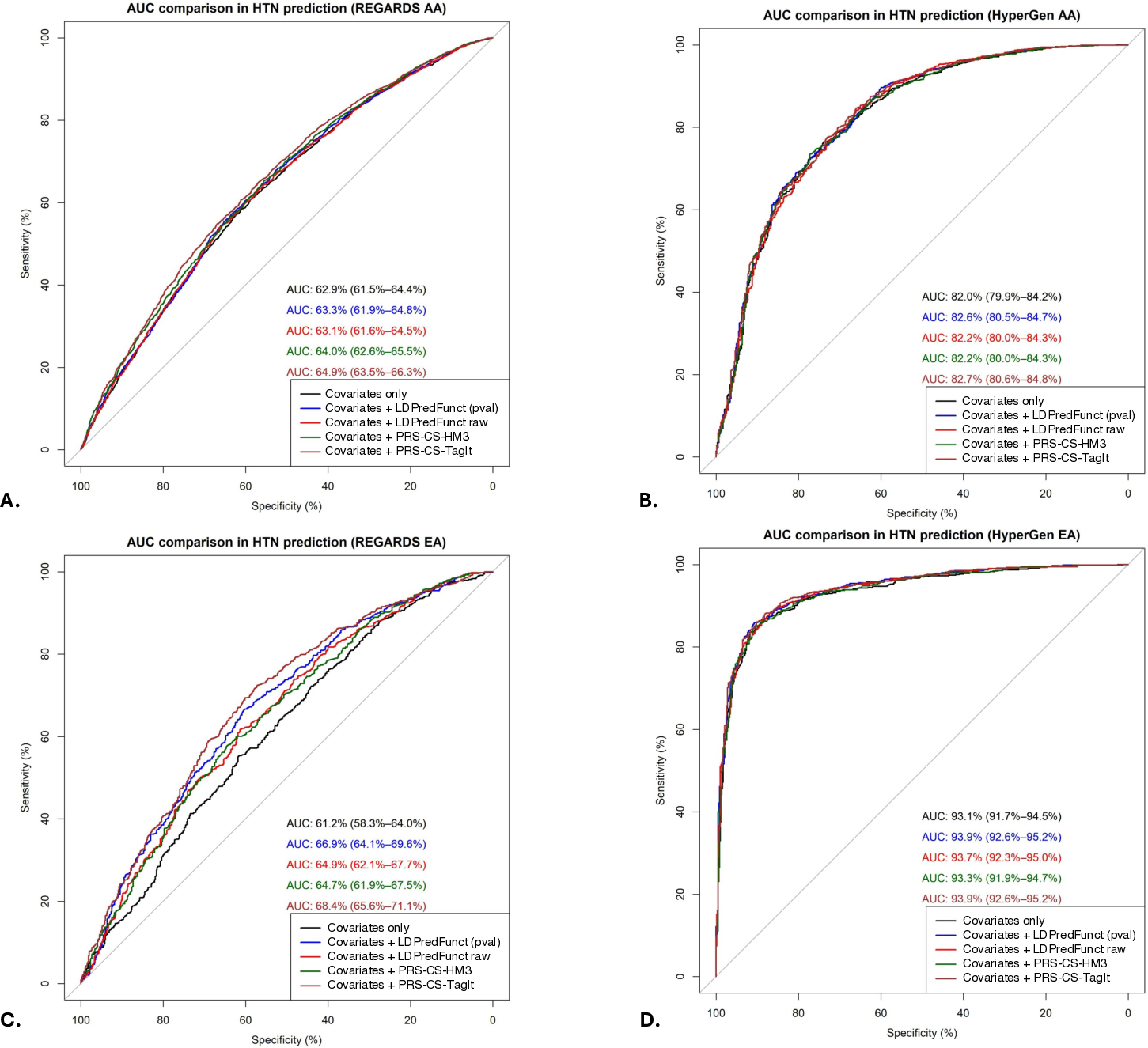

Supplement: S1 Fig — A) AUC comparisons in AA participants from REGARDS optimization cohort. B) AUC comparisons in AA participants from HyperGEN validation cohort. C) AUC comparisons in EA participants from REGARDS optimization cohort. D) AUC comparisons in EA participants from HyperGEN validation cohort. (TIF) [file pone.0353311.s001.tif]
