# Supplementary material for: Improving performance of polygenic risk scores for hypertension across two ancestry groups
Source: PLoS One. 2026 Jul 24;21(7):e0353311. doi: 10.1371/journal.pone.0353311 (PMC13399276; doi:10.1371/journal.pone.0353311)
Supplement: Attachment — Submitted filename: Plos_One_HTNreviewresponse-4_10.docx [file pone.0353311.s009.docx]

**Reviewer #1: The manuscript evaluates the impact of incorporating functional annotations and alternative LD reference panels on PRS performance for hypertension across EA and AA populations. Using two independent cohorts, the authors show that both functional priors and an expanded, multi-ancestry LD reference panel improve PRS performance, with larger gains observed in EA. Overall, the study addresses an important methodological question in PRS development and is suitable for publication. While some issues should be addressed with minor revisions.**

**1. The larger number of variants included in LDpred-funct scores compared to PRS-CS may explain some of the performance differences. Please clarify whether performance gains reflect functional annotation, variant density, or both. Consider including sensitivity analysis using matched SNP sets or acknowledge this limitation.**

For PRS-CS, the performance gains between the HM3 panel and the TagIt panel are due to SNP density. For LDpred-funct it’s harder to tell since there are many more SNPs (i.e., there are >3.5 million SNPs in the scores for LDpred-funct and >800K SNPs in the scores for PR-CS-HM3), making both SNP density and functional annotations potential contributors to the improvement. It is difficult to make these comparisons apples to apples, but we did take the suggestion for the proposed sensitivity analysis for LDpred-funct in HyperGEN. After checking the overlap between the SNPs included in the PRS-CS-HM3 score and LDpred-funct score, we found 756,481 SNPs in common for the AA strata and 788,583 SNPs for the EA strata. We ran the PRS metrics for the overlapping SNPs in each race strata (see results in bold in response Table below). Given that these LDpred-funct results are comparable to the primary results with many more SNPs, we suggest that the gains are primarily due to the functional annotations and less so due to the increased density of variants. We have added these results to the supplement (Supplementary Table 3) and added the following sentence to the methods, results, and discussion.

**Methods page 19, lines 428-431**: In a sensitivity analysis in HyperGEN, we restricted the SNP set for LDpred-funct to a set of variants overlapping with the PRS-CS-HM3 score (756,481 for AAs and 788,583 for EAs) to help determine if performance gains from LDpred-funct were influenced more by the increased count of variants or functional annotations.

**Results page 9, lines 176-177**: Results for the restricted SNP set for LDpred-funct are presented in Supplementary Table 3, showing comparable performance to the primary results.

**Discussion page 10, lines 209-215**: In our study, the scores created by LDpred-funct had many more variants (~3X more) than for PRS-CS making it hard to determine if performance gains were more influenced by SNP density or functional annotations. When we restricted LDpred-funct score performance to reflect only a set of SNPs overlapping with those present in the PRS-CS-HM3 score the results were slightly attenuated from the original results in each race strata. This suggests the majority of the performance gain for LDpred-funct may be coming from functional annotations as opposed to the increased density of variants in the score.

| **Response Document Table: HTN Results per 1 standard Deviation of the PRS** | | | | | | |
| --- | --- | --- | --- | --- | --- | --- |
| **Cohort** | **PRS Method** | **Phi**  **Parameter** | **OR (95% CI)** | **P** | **R^2^** | **AUC PRS** |
| REGARDS EAs | PRS-CS-HM3 | 1x10^-04*^ | 1.37 (1.23-1.53) | 1.02E-08 | 2.71E-02 | 0.58 |
|  | PRS-CS-TagIt | 1x10^-04*^ | 1.73 (1.54-1.94) | 4.48E-21 | 7.79E-02 | 0.64 |
|  | LDPred-funct | - | 1.51 (1.35-1.68) | 3.74E-13 | 4.46E-02 | 0.61 |
| HyperGEN EAs | PRS-CS-HM3 | 1x10^-04*^ | 1.40(1.07-1.82) | 1.19E-02 | 1.37E-02 | 0.56 |
|  | PRS-CS-TagIt | 1x10^-04*^ | 2.17 (1.65-2.85) | 3.00E-08 | 7.25E-02 | 0.61 |
|  | LDPred-funct | - | 2.14 (1.61-2.85) | 1.46E-07 | 6.03E-02 | 0.60 |
|  | **LDPred-funct (overlapping)** | **-** | **1.74 (1.33-2.28)** | **6.61E-05** | **3.12E-02** | **0.58** |
| REGARDS AAs | PRS-CS-HM3 | 1x10^-04*^ | 1.25 (1.19-1.32) | 1.39E-16 | 1.76E-02 | 0.56 |
|  | PRS-CS-TagIt | 1x10^-04*^ | 1.28 (1.21-1.35) | 5.36E-19 | 2.06E-02 | 0.57 |
|  | LDPred-funct | - | 1.32 (1.25-1.39) | 5.20E-24 | 2.66E-02 | 0.57 |
| HyperGEN AAs | PRS-CS-HM3 | 1x10^-04*^ | 1.20 (1.04-1.39) | 1.15E-02 | 6.70E-03 | 0.53 |
|  | PRS-CS-TagIt | 1x10^-04*^ | 1.47 (1.27-1.69) | 1.14E-07 | 2.94E-02 | 0.55 |
|  | LDPred-funct | - | 1.37 (1.19-1.58) | 1.76E-05 | 1.93E-02 | 0.52 |
|  | **LDPred-funct (overlapping)** | **-** | **1.37 (1.18-1.58)** | **2.43E-05** | **1.88E-02** | **0.54** |
| *Best model Phi in REGARDS, AA-African American, EA-European American, HM3-HapMap 3 LD Panel, TagIt-Tagging) It(erative) of SNPs LD Panel, AUC-Area Under the Curve | | | | | | |

**2. The methods clearly describe optimization of the PRS-CS phi parameter via grid search in the REGARDS cohort, with the best-performing phi for validation in HyperGEN. Please also clarify in the Results that phi was tuned exclusively in REGARDS and not re-optimized in HyperGEN and confirm whether phi optimization was performed separately by ancestry group.**

Thank you. Yes, the phi parameter was tuned exclusively in REGARDS. Phi optimization was performed separately for each ancestry group. We have confirmed this in the methods section.

**Methods page 17, lines 391-392**: Phi was tuned exclusively in REGARDS; phi optimization was performed separately by ancestry group.

**3. Other minor issues: inconsistent name of LD-Pred-funct vs LDPred-funct. Inconsistent R² formatting. Typo in CI in the Abstract.**

Thank you for pointing these errors out. We have updated the manuscript and supplemental material with regard to these issues.

**Reviewer #2: The authors have done good work benchmarking PRS performance across different models and ancestry groups. However, several issues need clarification or further consideration.**

**1. Authors should carefully check the numbers in the manuscript. For example,
a) The abstract reports “1,533 EA (58% with HTN)”, whereas the introduction reports “1,598 EA” (page 5, line 107).**

Thank you, we have updated the numbers for REGARDS EA on page 5 line 107, which was a typo.

**b) It was mentioned “variants from a 1000G phase 3 reference panel (including 1,217,312 variants)” in the methods (page 16, line 377), but “3.5M variants” in Table 2.**

To clarify, the difference is that the counts in Table 2 are the number of SNPs that make up the PRS. The count on page 16 was the count of variants in the LDpred-funct LD reference panel used to create the score. We have updated the column header in Table 2 to say “Variants in Final PRS”.

**2. It would be helpful to test LDpred-funct using different LD reference panels (e.g., TagIt vs. HM3) to allow a more direct comparison with PRS-CS and to better identify the effect of the LD panel.**

Given that the expanded reference panel includes SNPs not represented in the 1000 Genomes Phase 3 panel used by LDpred-funct, adapting it would require a more gradual approach involving: (a) annotating a broader set of variants, (b) reconstructing the LD structure for the full variant set, and (c) conducting stratified LD score regression to assess low-level LD (LLD) correlations with functional annotations. Due to the computational challenges of replacing the LD structure and revising the functional priors, we are unable to pursue this approach in the present paper, but we leave it for future work. We mentioned this point in the limitations.

**Discussion page 13, lines 276-279**. Due to the computational challenges of replacing the LD structure and revising the functional priors in LDpred-funct, we were unable to update the reference panel in that program which would have allowed a more direct comparison with PRS-CS.

**3. Due to the high variation of African ancestry among the AA individuals, it would be meaningful to stratify the AA group according to their African ancestry and further evaluate the performance of PRS within subgroups.**

Thank you for this good suggestion. We have stratified the results for REGARDS AAs as follows, and provided the results in the Table below, as well as the supplement. Results were very consistent across African Ancestry strata, but showed a slight improvement among participants with low African Ancestry. We have made the following changes to the manuscript.

Low African Ancestry (N=3,056): African admixture estimates < 0.8

Mid African Ancestry (N=3,168): 0.8 <= African admixture estimates < 0.9

High African Ancestry (N=2,231): African admixture estimates >= 0.9

| **Response Table: HTN Results per 1 standard Deviation of the PRS by African Ancestry** | | | | | | |
| --- | --- | --- | --- | --- | --- | --- |
| **Cohort** | **PRS Method** | **Parameter** | **OR (95% CI)** | **P** | **R^2^** | **AUC PRS** |
|  | PRS-CS-TagIt | 1x10^-04*^ | 1.28 (1.21-1.35) | 5.36E-19 | 2.06E-02 | 0.57 |
|  | PRS-CS-TagIt-Low | 1x10^-04*^ | 1.42 (1.30-1.55) | 3.17E-14 | 3.90E-02 | 0.59 |
|  | PRS-CS-TagIt-Mid | 1x10^-04*^ | 1.28 (1.17-1.40) | 6.15E-08 | 2.11E-02 | 0.56 |
|  | PRS-CS-TagIt-High | 1x10^-04*^ | 1.28 (1.15-1.41) | 4.38E-06 | 2.21E-02 | 0.56 |
|  | LDPred-funct | - | 1.32 (1.25-1.39) | 5.20E-24 | 2.66E-02 | 0.57 |
|  | LDPred-funct-Low | - | 1.37 (1.25-1.50) | 5.96E-12 | 3.19E-02 | 0.57 |
|  | LDPred-funct-Mid | - | 1.27 (1.16-39) | 1.09E-07 | 2.03E-02 | 0.56 |
|  | LDPred-funct-High | - | 1.35 (1.22-1.50) | 1.65E-08 | 3.37E-02 | 0.58 |
| *Best model Phi REGARDS, AA-African American, EA-European American, HM3-HapMap 3 LD Panel, TagIt-Tag(ging) It(erative) of SNPs LD Panel, AUC-Area Under the Curve | | | | | | |

**Methods page 19, lines 431-436**: In an additional sensitivity analysis, the global African ancestry percentage was calculated for each REGARDS AA participant using Admixture version 1.3.0 software running an unsupervised method for k = 2 ancestral populations as described by Parcha et al. We divided the participants at the following thresholds: low (<0.8, N=3,056), mid (0.8 <= African admixture estimates < 0.9, N=3,168), high (>= 0.9, N=2,231) and calculated the same PRS metrics as for the main results.

**Results page 9, lines 177-180**: In a second sensitivity analysis Supplementary Table 4 reports the results for PRS-CS-TagIt and LDpred-funct stratified by African Ancestry percentage. The results are largely consistent with the main result but do show a trend for better performance among participants with low African ancestry.

**4. How about incorporating the local ancestry as a covariate while analyzing the AA group? At least, the potential role of local ancestry should be discussed in the manuscript.**

Thank you for this suggestion. Given that the PRS are global genomic scores (i.e., weighted average of all SNPs), we were unable to include the effect of local ancestry at the component SNPs using the tools employed. Addressing this would have necessitated rewriting the PRS-CS and LDpred-Funct code and rerunning the full set of analyses from scratch, including both training and testing phases. This falls outside the scope of the current manuscript and will be explored in future research. We reviewed the paper on modeling local ancestry in PRS development, namely, GAUDI (Genetic Ancestry Utilization in polygenic risk scores for aDmixed Individuals). While this method did outperform other standard methods (e.g., PRSCSx) for some traits it did not for HTN or SBP (Sun *et al.*, Nature 2024). We have mentioned this as a limitation in the discussion.

**Discussion page 13, lines 279-282**: Finally, the methods we employed did not incorporate the effects of local ancestry at each contributing SNP as a potential modifying effect in AAs which would be interesting to evaluate in future studies. However, our sensitivity analysis in REGARDS showed results were comparable across strata of African.
