# Supplementary material for: Improving performance of polygenic risk scores for hypertension across two ancestry groups
Source: PLoS One. 2026 Jul 24;21(7):e0353311. doi: 10.1371/journal.pone.0353311 (PMC13399276; doi:10.1371/journal.pone.0353311)
Supplement: Attachment — Submitted filename: Reviewerresponse2_5_18.docx [file pone.0353311.s010.docx]

Reviewer #2: The authors have addressed R1 comments well. However, several issues remain and should be clarified:

1. The abstract reports “OR = 1.40; 95% CI 10.8–1.82” (line 21), which was flagged by Reviewer 1. I believe that the authors should carefully verify all numerical values throughout the manuscript.

Thank you, we have updated this typo. We have also cross-checked text with tables throughout.

Abstract lines 20-21: “The magnitude of the OR per SD for HTN was also higher for PRS-CS-TagIt OR=2.17 (95% CI 1.65-2.85, p=3.0*10^-8^) and LDpred-funct OR=2.14 (95% CI 1.61-2.85, p=1.46*10^-7^) versus PRS-CS-HM3 (OR=1.40; 95% CI 1.07-1.82, p=1.19*10^-2^).”

2. The naming of “LDpred-funct” remains inconsistent. In fact, additional inconsistencies have been introduced in the revised version.

We have double checked the manuscript. It is written as ‘LDpred-funct’ throughout. We have now updated the formatting in the figures as well.

3. The LD panel for LDpred-funct is now changed to “HM3,” whereas the Methods section (lines 394–395) states that LDpred-funct uses a 1000 Genomes Phase 3 reference panel.

We updated it to 1000 Genomes Phase 3 in both Table 2 and the methods lines 395-397. We have also corrected the right side panel in Figure 1.

4. The restricted SNP-set sensitivity analysis for LDpred-funct (Supplementary Table 3) deserves further discussion. In EAs, the overlapping-SNP LDpred-funct score (OR = 1.74) still outperforms PRS-CS-HM3 (OR = 1.40), but is substantially attenuated compared to the full LDpred-funct score (OR = 2.14). This pattern suggests that while functional annotations contribute meaningfully, SNP density also plays an important role.

Thank you for the comment. In response, we have tweaked our discussion point in this favor.

Discussion page 10, lines 209-216: In our study, the scores created by LDpred-funct had many more variants (~3X more) than for PRS-CS making it hard to determine if performance gains were more influenced by SNP density or functional annotations. When we restricted LDpred-funct score performance to reflect only a set of SNPs overlapping with those present in the PRS-CS-HM3 score the results were slightly attenuated from the original results in each race strata. This suggests the majority of the performance gain for LDpred-funct may be coming from functional annotations though the increased density of variants in the score is also playing a role at least in the EA strata.
