# Supplementary material for: Improving performance of polygenic risk scores for hypertension across two ancestry groups
Source: PLoS One. 2026 Jul 24;21(7):e0353311. doi: 10.1371/journal.pone.0353311 (PMC13399276; doi:10.1371/journal.pone.0353311)
Supplement: Peer review history

# Author comment: Author response to Decision Letter 0

**DOI:** 10.1371/journal.pone.0353311.r001

**Transfer Alert**

This paper was transferred from another journal. As a result, its full editorial history (including decision letters, peer reviews and author responses) may not be present.

11 Dec 2025

# Peer review: Decision Letter 0

**DOI:** 10.1371/journal.pone.0353311.r002

**Authors:** Hoh Boon-Peng

25 Feb 2026

PONE-D-25-63368

Improving performance of polygenic risk scores for hypertension across two ancestry groups.

PLOS One

Dear Dr. Irvin,

Thank you for submitting your manuscript to PLOS ONE. After careful consideration, we feel that it has merit but does not fully meet PLOS ONE’s publication criteria as it currently stands. Therefore, we invite you to submit a revised version of the manuscript that addresses the points raised during the review process.

Please submit your revised manuscript by Apr 11 2026 11:59PM. If you will need more time than this to complete your revisions, please reply to this message or contact the journal office at plosone@plos.org. When you're ready to submit your revision, log on to https://www.editorialmanager.com/pone/ and select the 'Submissions Needing Revision' folder to locate your manuscript file.

Please include the following items when submitting your revised manuscript:

- A letter that responds to each point raised by the academic editor and reviewer(s). You should upload this letter as a separate file labeled 'Response to Reviewers'.
- A marked-up copy of your manuscript that highlights changes made to the original version. You should upload this as a separate file labeled 'Revised Manuscript with Track Changes'.
- An unmarked version of your revised paper without tracked changes. You should upload this as a separate file labeled 'Manuscript'.

If you would like to make changes to your financial disclosure, please include your updated statement in your cover letter. Guidelines for resubmitting your figure files are available below the reviewer comments at the end of this letter.

If applicable, we recommend that you deposit your laboratory protocols in protocols.io to enhance the reproducibility of your results. Protocols.io assigns your protocol its own identifier (DOI) so that it can be cited independently in the future. For instructions see: https://journals.plos.org/plosone/s/submission-guidelines#loc-laboratory-protocols. Additionally, PLOS ONE offers an option for publishing peer-reviewed Lab Protocol articles, which describe protocols hosted on protocols.io. Read more information on sharing protocols at https://plos.org/protocols?utm_medium=editorial-email&utm_source=authorletters&utm_campaign=protocols.

We look forward to receiving your revised manuscript.

Kind regards,

Hoh Boon-Peng, PhD

Academic Editor

PLOS One

Journal Requirements:

When submitting your revision, we need you to address these additional requirements.

1.Please ensure that your manuscript meets PLOS ONE's style requirements, including those for file naming. The PLOS ONE style templates can be found at

https://journals.plos.org/plosone/s/file?id=wjVg/PLOSOne_formatting_sample_main_body.pdf and

https://journals.plos.org/plosone/s/file?id=ba62/PLOSOne_formatting_sample_title_authors_affiliations.pdf

2. Please note that PLOS One has specific guidelines on code sharing for submissions in which author-generated code underpins the findings in the manuscript. In these cases, we expect all author-generated code to be made available without restrictions upon publication of the work. Please review our guidelines at https://journals.plos.org/plosone/s/materials-and-software-sharing#loc-sharing-code and ensure that your code is shared in a way that follows best practice and facilitates reproducibility and reuse.

3. Please note that funding information should not appear in any section or other areas of your manuscript. We will only publish funding information present in the Funding Statement section of the online submission form. Please remove any funding-related text from the manuscript.

4. We note that the grant information you provided in the ‘Funding Information’ and ‘Financial Disclosure’ sections do not match.

When you resubmit, please ensure that you provide the correct grant numbers for the awards you received for your study in the ‘Funding Information’ section.

5. Please note that your Data Availability Statement is currently missing the repository name and/or the DOI/accession number of each dataset OR a direct link to access each database. If your manuscript is accepted for publication, you will be asked to provide these details on a very short timeline. We therefore suggest that you provide this information now, though we will not hold up the peer review process if you are unable.

6. Please include captions for your Supporting Information files at the end of your manuscript, and update any in-text citations to match accordingly. Please see our Supporting Information guidelines for more information: http://journals.plos.org/plosone/s/supporting-information.

7. If the reviewer comments include a recommendation to cite specific previously published works, please review and evaluate these publications to determine whether they are relevant and should be cited. There is no requirement to cite these works unless the editor has indicated otherwise.

Additional Editor Comments:

The reviewers have raised several comments, primarily on the LD-fred and the sample stratification.

Authors are invited to response the reviewers point carefully and resubmit the manuscript.

[Note: HTML markup is below. Please do not edit.]

Reviewers' comments:

Reviewer's Responses to Questions

**Comments to the Author**

1. Is the manuscript technically sound, and do the data support the conclusions?

The manuscript must describe a technically sound piece of scientific research with data that supports the conclusions. Experiments must have been conducted rigorously, with appropriate controls, replication, and sample sizes. The conclusions must be drawn appropriately based on the data presented.

Reviewer #1: Yes

Reviewer #2: Yes

**********

2. Has the statistical analysis been performed appropriately and rigorously?

Reviewer #1: Yes

Reviewer #2: Yes

**********

3. Have the authors made all data underlying the findings in their manuscript fully available?

The PLOS Data policy requires authors to make all data underlying the findings described in their manuscript fully available without restriction, with rare exception (please refer to the Data Availability Statement in the manuscript PDF file). The data should be provided as part of the manuscript or its supporting information, or deposited to a public repository. For example, in addition to summary statistics, the data points behind means, medians and variance measures should be available. If there are restrictions on publicly sharing data—e.g. participant privacy or use of data from a third party—those must be specified.

Reviewer #1: Yes

Reviewer #2: Yes

**********

4. Is the manuscript presented in an intelligible fashion and written in standard English?

PLOS ONE does not copyedit accepted manuscripts, so the language in submitted articles must be clear, correct, and unambiguous. Any typographical or grammatical errors should be corrected at revision, so please note any specific errors here.

Reviewer #1: Yes

Reviewer #2: Yes

**********

5. Review Comments to the Author

Please use the space provided to explain your answers to the questions above. You may also include additional comments for the author, including concerns about dual publication, research ethics, or publication ethics. (Please upload your review as an attachment if it exceeds 20,000 characters)

Reviewer #1: The manuscript evaluates the impact of incorporating functional annotations and alternative LD reference panels on PRS performance for hypertension across EA and AA populations. Using two independent cohorts, the authors show that both functional priors and an expanded, multi-ancestry LD reference panel improve PRS performance, with larger gains observed in EA. Overall, the study addresses an important methodological question in PRS development and is suitable for publication. While some issues should be addressed with minor revisions.

1. The larger number of variants included in LDpred-funct scores compared to PRS-CS may explain some of the performance differences. Please clarify whether performance gains reflect functional annotation, variant density, or both. Consider including sensitivity analysis using matched SNP sets or acknowledge this limitation.

2. The methods clearly describe optimization of the PRS-CS phi parameter via grid search in the REGARDS cohort, with the best-performing phi for validation in HyperGEN. Please also clarify in the Results that phi was tuned exclusively in REGARDS and not re-optimized in HyperGEN and confirm whether phi optimization was performed separately by ancestry group.

3. Other minor issues: inconsistent name of LD-Pred-funct vs LDPred-funct. Inconsistent R² formatting. Typo in CI in the Abstract.

Reviewer #2: The authors have done good work benchmarking PRS performance across different models and ancestry groups. However, several issues need clarification or further consideration.

1. Authors should carefully check the numbers in the manuscript. For example,

a) The abstract reports “1,533 EA (58% with HTN)”, whereas the introduction reports “1,598 EA” (page 5, line 107).

b) It was mentioned “variants from a 1000G phase 3 reference panel (including 1,217,312 variants)” in the methods (page 16, line 377), but “3.5M variants” in Table 2.

2. It would be helpful to test LDpred-funct using different LD reference panels (e.g., TagIt vs. HM3) to allow a more direct comparison with PRS-CS and to better identify the effect of the LD panel.

3. Due to the high variation of African ancestry among the AA individuals, it would be meaningful to stratify the AA group according to their African ancestry and further evaluate the performance of PRS within subgroups.

4. How about incorporating the local ancestry as a covariate while analyzing the AA group ? At least, the potential role of local ancestry should be discussed in the manuscript.

**********

6. PLOS authors have the option to publish the peer review history of their article (what does this mean?). If published, this will include your full peer review and any attached files.

If you choose “no”, your identity will remain anonymous but your review may still be made public.

**Do you want your identity to be public for this peer review?**  For information about this choice, including consent withdrawal, please see our Privacy Policy.

Reviewer #1: No

Reviewer #2: No

**********

[NOTE: If reviewer comments were submitted as an attachment file, they will be attached to this email and accessible via the submission site. Please log into your account, locate the manuscript record, and check for the action link "View Attachments". If this link does not appear, there are no attachment files.]

To ensure your figures meet our technical requirements, please review our figure guidelines: https://journals.plos.org/plosone/s/figures

You may also use PLOS’s free figure tool, NAAS, to help you prepare publication quality figures: https://journals.plos.org/plosone/s/figures#loc-tools-for-figure-preparation.

NAAS will assess whether your figures meet our technical requirements by comparing each figure against our figure specifications.

# Author comment: Author response to Decision Letter 1

**DOI:** 10.1371/journal.pone.0353311.r003

14 Apr 2026

Reviewer #1: The manuscript evaluates the impact of incorporating functional annotations and alternative LD reference panels on PRS performance for hypertension across EA and AA populations. Using two independent cohorts, the authors show that both functional priors and an expanded, multi-ancestry LD reference panel improve PRS performance, with larger gains observed in EA. Overall, the study addresses an important methodological question in PRS development and is suitable for publication. While some issues should be addressed with minor revisions.

1. The larger number of variants included in LDpred-funct scores compared to PRS-CS may explain some of the performance differences. Please clarify whether performance gains reflect functional annotation, variant density, or both. Consider including sensitivity analysis using matched SNP sets or acknowledge this limitation.

For PRS-CS, the performance gains between the HM3 panel and the TagIt panel are due to SNP density. For LDpred-funct it’s harder to tell since there are many more SNPs (i.e., there are >3.5 million SNPs in the scores for LDpred-funct and >800K SNPs in the scores for PR-CS-HM3), making both SNP density and functional annotations potential contributors to the improvement. It is difficult to make these comparisons apples to apples, but we did take the suggestion for the proposed sensitivity analysis for LDpred-funct in HyperGEN. After checking the overlap between the SNPs included in the PRS-CS-HM3 score and LDpred-funct score, we found 756,481 SNPs in common for the AA strata and 788,583 SNPs for the EA strata. We ran the PRS metrics for the overlapping SNPs in each race strata (see results in bold in response Table in attached document). Given that these LDpred-funct results are comparable to the primary results with many more SNPs, we suggest that the gains are primarily due to the functional annotations and less so due to the increased density of variants. We have added these results to the supplement (Supplementary Table 3) and added the following sentence to the methods, results, and discussion.

Methods page 19, lines 428-431: In a sensitivity analysis in HyperGEN, we restricted the SNP set for LDpred-funct to a set of variants overlapping with the PRS-CS-HM3 score (756,481 for AAs and 788,583 for EAs) to help determine if performance gains from LDpred-funct were influenced more by the increased count of variants or functional annotations.

Results page 9, lines 176-177: Results for the restricted SNP set for LDpred-funct are presented in Supplementary Table 3, showing comparable performance to the primary results.

Discussion page 10, lines 209-215: In our study, the scores created by LDpred-funct had many more variants (~3X more) than for PRS-CS making it hard to determine if performance gains were more influenced by SNP density or functional annotations. When we restricted LDpred-funct score performance to reflect only a set of SNPs overlapping with those present in the PRS-CS-HM3 score the results were slightly attenuated from the original results in each race strata. This suggests the majority of the performance gain for LDpred-funct may be coming from functional annotations as opposed to the increased density of variants in the score.

2. The methods clearly describe optimization of the PRS-CS phi parameter via grid search in the REGARDS cohort, with the best-performing phi for validation in HyperGEN. Please also clarify in the Results that phi was tuned exclusively in REGARDS and not re-optimized in HyperGEN and confirm whether phi optimization was performed separately by ancestry group.

Thank you. Yes, the phi parameter was tuned exclusively in REGARDS. Phi optimization was performed separately for each ancestry group. We have confirmed this in the methods section.

Methods page 17, lines 391-392: Phi was tuned exclusively in REGARDS; phi optimization was performed separately by ancestry group.

3. Other minor issues: inconsistent name of LD-Pred-funct vs LDPred-funct. Inconsistent R² formatting. Typo in CI in the Abstract.

Thank you for pointing these errors out. We have updated the manuscript and supplemental material with regard to these issues.

Reviewer #2: The authors have done good work benchmarking PRS performance across different models and ancestry groups. However, several issues need clarification or further consideration.

1. Authors should carefully check the numbers in the manuscript. For example,

a) The abstract reports “1,533 EA (58% with HTN)”, whereas the introduction reports “1,598 EA” (page 5, line 107).

Thank you, we have updated the numbers for REGARDS EA on page 5 line 107, which was a typo.

b) It was mentioned “variants from a 1000G phase 3 reference panel (including 1,217,312 variants)” in the methods (page 16, line 377), but “3.5M variants” in Table 2.

To clarify, the difference is that the counts in Table 2 are the number of SNPs that make up the PRS. The count on page 16 was the count of variants in the LDpred-funct LD reference panel used to create the score. We have updated the column header in Table 2 to say “Variants in Final PRS”.

2. It would be helpful to test LDpred-funct using different LD reference panels (e.g., TagIt vs. HM3) to allow a more direct comparison with PRS-CS and to better identify the effect of the LD panel.

Given that the expanded reference panel includes SNPs not represented in the 1000 Genomes Phase 3 panel used by LDpred-funct, adapting it would require a more gradual approach involving: (a) annotating a broader set of variants, (b) reconstructing the LD structure for the full variant set, and (c) conducting stratified LD score regression to assess low-level LD (LLD) correlations with functional annotations. Due to the computational challenges of replacing the LD structure and revising the functional priors, we are unable to pursue this approach in the present paper, but we leave it for future work. We mentioned this point in the limitations.

Discussion page 13, lines 276-279. Due to the computational challenges of replacing the LD structure and revising the functional priors in LDpred-funct, we were unable to update the reference panel in that program which would have allowed a more direct comparison with PRS-CS.

3. Due to the high variation of African ancestry among the AA individuals, it would be meaningful to stratify the AA group according to their African ancestry and further evaluate the performance of PRS within subgroups.

Thank you for this good suggestion. We have stratified the results for REGARDS AAs as follows, and provided the results in the Table in the response attachment, as well as the supplement. Results were very consistent across African Ancestry strata, but showed a slight improvement among participants with low African Ancestry. We have made the following changes to the manuscript.

Low African Ancestry (N=3,056): African admixture estimates < 0.8

Mid African Ancestry (N=3,168): 0.8 <= African admixture estimates < 0.9

High African Ancestry (N=2,231): African admixture estimates >= 0.9

Methods page 19, lines 431-436: In an additional sensitivity analysis, the global African ancestry percentage was calculated for each REGARDS AA participant using Admixture version 1.3.0 software running an unsupervised method for k = 2 ancestral populations as described by Parcha et al. We divided the participants at the following thresholds: low (<0.8, N=3,056), mid (0.8 <= African admixture estimates < 0.9, N=3,168), high (>= 0.9, N=2,231) and calculated the same PRS metrics as for the main results.

Results page 9, lines 177-180: In a second sensitivity analysis Supplementary Table 4 reports the results for PRS-CS-TagIt and LDpred-funct stratified by African Ancestry percentage. The results are largely consistent with the main result but do show a trend for better performance among participants with low African ancestry.

4. How about incorporating the local ancestry as a covariate while analyzing the AA group? At least, the potential role of local ancestry should be discussed in the manuscript.

Thank you for this suggestion. Given that the PRS are global genomic scores (i.e., weighted average of all SNPs), we were unable to include the effect of local ancestry at the component SNPs using the tools employed. Addressing this would have necessitated rewriting the PRS-CS and LDpred-Funct code and rerunning the full set of analyses from scratch, including both training and testing phases. This falls outside the scope of the current manuscript and will be explored in future research. We reviewed the paper on modeling local ancestry in PRS development, namely, GAUDI (Genetic Ancestry Utilization in polygenic risk scores for aDmixed Individuals). While this method did outperform other standard methods (e.g., PRSCSx) for some traits it did not for HTN or SBP (Sun et al., Nature 2024). We have mentioned this as a limitation in the discussion.

Discussion page 13, lines 279-282: Finally, the methods we employed did not incorporate the effects of local ancestry at each contributing SNP as a potential modifying effect in AAs which would be interesting to evaluate in future studies. However, our sensitivity analysis in REGARDS showed results were comparable across strata of African.

Attachment Submitted filename: Plos_One_HTNreviewresponse-4_10.docx

# Peer review: Decision Letter 1

**DOI:** 10.1371/journal.pone.0353311.r004

**Authors:** Hoh Boon-Peng

18 May 2026

PONE-D-25-63368R1

Improving performance of polygenic risk scores for hypertension across two ancestry groups.

PLOS One

Dear Dr. Irvin,

Thank you for submitting your manuscript to PLOS ONE. After careful consideration, we feel that it has merit but does not fully meet PLOS ONE’s publication criteria as it currently stands. Therefore, we invite you to submit a revised version of the manuscript that addresses the points raised during the review process.

==============================

The manuscript is generally accepted by the reviewers, except for a few minor comments which I invite the authors to address the comments accordingly.

==============================

Please submit your revised manuscript by Jul 02 2026 11:59PM. If you will need more time than this to complete your revisions, please reply to this message or contact the journal office at plosone@plos.org. When you're ready to submit your revision, log on to https://www.editorialmanager.com/pone/ and select the 'Submissions Needing Revision' folder to locate your manuscript file.

Please include the following items when submitting your revised manuscript:

- A letter that responds to each point raised by the academic editor and reviewer(s). You should upload this letter as a separate file labeled 'Response to Reviewers'.
- A marked-up copy of your manuscript that highlights changes made to the original version. You should upload this as a separate file labeled 'Revised Manuscript with Track Changes'.
- An unmarked version of your revised paper without tracked changes. You should upload this as a separate file labeled 'Manuscript'.

If you would like to make changes to your financial disclosure, please include your updated statement in your cover letter. Guidelines for resubmitting your figure files are available below the reviewer comments at the end of this letter.

If applicable, we recommend that you deposit your laboratory protocols in protocols.io to enhance the reproducibility of your results. Protocols.io assigns your protocol its own identifier (DOI) so that it can be cited independently in the future. For instructions see: https://journals.plos.org/plosone/s/submission-guidelines#loc-laboratory-protocols. Additionally, PLOS ONE offers an option for publishing peer-reviewed Lab Protocol articles, which describe protocols hosted on protocols.io. Read more information on sharing protocols at https://plos.org/protocols?utm_medium=editorial-email&utm_source=authorletters&utm_campaign=protocols.

As the corresponding author, your ORCID iD is verified in the submission system and will appear in the published article. PLOS supports the use of ORCID, and we encourage all coauthors to register for an ORCID iD and use it as well. Please encourage your coauthors to verify their ORCID iD within the submission system before final acceptance, as unverified ORCID iDs will not appear in the published article. *Only*  the individual author can complete the verification step; PLOS staff *cannot*  verify ORCID iDs on behalf of authors.

We look forward to receiving your revised manuscript.

Kind regards,

Hoh Boon-Peng, PhD

Academic Editor

PLOS One

Journal Requirements:

If the reviewer comments include a recommendation to cite specific previously published works, please review and evaluate these publications to determine whether they are relevant and should be cited. There is no requirement to cite these works unless the editor has indicated otherwise.

Please review your reference list to ensure that it is complete and correct. If you have cited papers that have been retracted, please include the rationale for doing so in the manuscript text, or remove these references and replace them with relevant current references. Any changes to the reference list should be mentioned in the rebuttal letter that accompanies your revised manuscript. If you need to cite a retracted article, indicate the article’s retracted status in the References list and also include a citation and full reference for the retraction notice.

Additional Editor Comments:

The manuscript is generally accepted by the reviewers, except for a few minor comments which I invite the authors to address the comments accordingly.

[Note: HTML markup is below. Please do not edit.]

Reviewers' comments:

Reviewer's Responses to Questions

**Comments to the Author**

1. If the authors have adequately addressed your comments raised in a previous round of review and you feel that this manuscript is now acceptable for publication, you may indicate that here to bypass the “Comments to the Author” section, enter your conflict of interest statement in the “Confidential to Editor” section, and submit your "Accept" recommendation.

Reviewer #1: All comments have been addressed

Reviewer #2: (No Response)

**********

2. Is the manuscript technically sound, and do the data support the conclusions?

The manuscript must describe a technically sound piece of scientific research with data that supports the conclusions. Experiments must have been conducted rigorously, with appropriate controls, replication, and sample sizes. The conclusions must be drawn appropriately based on the data presented.

Reviewer #1: Yes

Reviewer #2: Yes

**********

3. Has the statistical analysis been performed appropriately and rigorously?

Reviewer #1: Yes

Reviewer #2: Yes

**********

4. Have the authors made all data underlying the findings in their manuscript fully available?

The PLOS Data policy requires authors to make all data underlying the findings described in their manuscript fully available without restriction, with rare exception (please refer to the Data Availability Statement in the manuscript PDF file). The data should be provided as part of the manuscript or its supporting information, or deposited to a public repository. For example, in addition to summary statistics, the data points behind means, medians and variance measures should be available. If there are restrictions on publicly sharing data—e.g. participant privacy or use of data from a third party—those must be specified.

Reviewer #1: Yes

Reviewer #2: Yes

**********

5. Is the manuscript presented in an intelligible fashion and written in standard English?

PLOS ONE does not copyedit accepted manuscripts, so the language in submitted articles must be clear, correct, and unambiguous. Any typographical or grammatical errors should be corrected at revision, so please note any specific errors here.

Reviewer #1: Yes

Reviewer #2: Yes

**********

6. Review Comments to the Author

Please use the space provided to explain your answers to the questions above. You may also include additional comments for the author, including concerns about dual publication, research ethics, or publication ethics. (Please upload your review as an attachment if it exceeds 20,000 characters)

Reviewer #1: The authors have addressed the points raised in my first review, added sensitivity analysis and corrected typos. I have no further concerns. I recommend acceptance without further revision.

Reviewer #2: The authors have addressed R1 comments well. However, several issues remain and should be clarified:

1. The abstract reports “OR = 1.40; 95% CI 10.8–1.82” (line 21), which was flagged by Reviewer 1. I believe that the authors should carefully verify all numerical values throughout the manuscript.

2. The naming of “LDpred-funct” remains inconsistent. In fact, additional inconsistencies have been introduced in the revised version.

3. The LD panel for LDpred-funct is now changed to “HM3,” whereas the Methods section (lines 394–395) states that LDpred-funct uses a 1000 Genomes Phase 3 reference panel.

4. The restricted SNP-set sensitivity analysis for LDpred-funct (Supplementary Table 3) deserves further discussion. In EAs, the overlapping-SNP LDpred-funct score (OR = 1.74) still outperforms PRS-CS-HM3 (OR = 1.40), but is substantially attenuated compared to the full LDpred-funct score (OR = 2.14). This pattern suggests that while functional annotations contribute meaningfully, SNP density also plays an important role.

**********

7. PLOS authors have the option to publish the peer review history of their article (what does this mean?). If published, this will include your full peer review and any attached files.

If you choose “no”, your identity will remain anonymous but your review may still be made public.

**Do you want your identity to be public for this peer review?**  For information about this choice, including consent withdrawal, please see our Privacy Policy.

Reviewer #1: No

Reviewer #2: No

**********

[NOTE: If reviewer comments were submitted as an attachment file, they will be attached to this email and accessible via the submission site. Please log into your account, locate the manuscript record, and check for the action link "View Attachments". If this link does not appear, there are no attachment files.]

To ensure your figures meet our technical requirements, please review our figure guidelines: https://journals.plos.org/plosone/s/figures

You may also use PLOS’s free figure tool, NAAS, to help you prepare publication quality figures: https://journals.plos.org/plosone/s/figures#loc-tools-for-figure-preparation.

NAAS will assess whether your figures meet our technical requirements by comparing each figure against our figure specifications.

# Author comment: Author response to Decision Letter 2

**DOI:** 10.1371/journal.pone.0353311.r005

27 May 2026

Reviewer #2: The authors have addressed R1 comments well. However, several issues remain and should be clarified:

1. The abstract reports “OR = 1.40; 95% CI 10.8–1.82” (line 21), which was flagged by Reviewer 1. I believe that the authors should carefully verify all numerical values throughout the manuscript.

Thank you, we have updated this typo. We have also cross-checked text with tables throughout.

Abstract lines 20-21: “The magnitude of the OR per SD for HTN was also higher for PRS-CS-TagIt OR=2.17 (95% CI 1.65-2.85, p=3.0*10-8) and LDpred-funct OR=2.14 (95% CI 1.61-2.85, p=1.46*10-7) versus PRS-CS-HM3 (OR=1.40; 95% CI 1.07-1.82, p=1.19*10-2).”

2. The naming of “LDpred-funct” remains inconsistent. In fact, additional inconsistencies have been introduced in the revised version.

We have double checked the manuscript. It is written as ‘LDpred-funct’ throughout. We have now updated the formatting in the figures as well.

3. The LD panel for LDpred-funct is now changed to “HM3,” whereas the Methods section (lines 394–395) states that LDpred-funct uses a 1000 Genomes Phase 3 reference panel.

We updated it to 1000 Genomes Phase 3 in both Table 2 and the methods lines 395-397. We have also corrected the right side panel in Figure 1.

4. The restricted SNP-set sensitivity analysis for LDpred-funct (Supplementary Table 3) deserves further discussion. In EAs, the overlapping-SNP LDpred-funct score (OR = 1.74) still outperforms PRS-CS-HM3 (OR = 1.40), but is substantially attenuated compared to the full LDpred-funct score (OR = 2.14). This pattern suggests that while functional annotations contribute meaningfully, SNP density also plays an important role.

Thank you for the comment. In response, we have tweaked our discussion point in this favor.

Discussion page 10, lines 209-216: In our study, the scores created by LDpred-funct had many more variants (~3X more) than for PRS-CS making it hard to determine if performance gains were more influenced by SNP density or functional annotations. When we restricted LDpred-funct score performance to reflect only a set of SNPs overlapping with those present in the PRS-CS-HM3 score the results were slightly attenuated from the original results in each race strata. This suggests the majority of the performance gain for LDpred-funct may be coming from functional annotations though the increased density of variants in the score is also playing a role at least in the EA strata.

Attachment Submitted filename: Reviewerresponse2_5_18.docx

# Editor report: Decision Letter 2

**DOI:** 10.1371/journal.pone.0353311.r006

**Authors:** Hoh Boon-Peng

22 Jun 2026

Improving performance of polygenic risk scores for hypertension across two ancestry groups.

PONE-D-25-63368R2

Dear Dr. Irvin,

We’re pleased to inform you that your manuscript has been judged scientifically suitable for publication and will be formally accepted for publication once it meets all outstanding technical requirements.

Within one week, you’ll receive an e-mail detailing the required amendments. When these have been addressed, you’ll receive a formal acceptance letter and your manuscript will be scheduled for publication.

An invoice will be generated when your article is formally accepted. Please note, if your institution has a publishing partnership with PLOS and your article meets the relevant criteria, all or part of your publication costs will be covered. Please make sure your user information is up-to-date by logging into Editorial Manager at Editorial Manager® and clicking the ‘Update My Information' link at the top of the page. For questions related to billing, please contact billing support.

If your institution or institutions have a press office, please notify them about your upcoming paper to help maximize its impact. If they’ll be preparing press materials, please inform our press team as soon as possible -- no later than 48 hours after receiving the formal acceptance. Your manuscript will remain under strict press embargo until 2 pm Eastern Time on the date of publication. For more information, please contact onepress@plos.org.

Kind regards,

Hoh Boon-Peng, PhD

Academic Editor

PLOS One

Additional Editor Comments (optional):

Reviewers' comments:

# Editor report: Acceptance letter

**DOI:** 10.1371/journal.pone.0353311.r007

**Authors:** Hoh Boon-Peng

PONE-D-25-63368R2

PLOS One

Dear Dr. Irvin,

I'm pleased to inform you that your manuscript has been deemed suitable for publication in PLOS One. Congratulations! Your manuscript is now being handed over to our production team.

At this stage, our production department will prepare your paper for publication. This includes ensuring the following:

* All references, tables, and figures are properly cited

* All relevant supporting information is included in the manuscript submission,

* There are no issues that prevent the paper from being properly typeset

You will receive further instructions from the production team, including instructions on how to review your proof when it is ready. Please keep in mind that we are working through a large volume of accepted articles, so please give us a few days to review your paper and let you know the next and final steps.

Lastly, if your institution or institutions have a press office, please let them know about your upcoming paper now to help maximize its impact. If they'll be preparing press materials, please inform our press team within the next 48 hours. Your manuscript will remain under strict press embargo until 2 pm Eastern Time on the date of publication. For more information, please contact onepress@plos.org.

You will receive an invoice from PLOS for your publication fee after your manuscript has reached the completed accept phase. If you receive an email requesting payment before acceptance or for any other service, this may be a phishing scheme. Learn how to identify phishing emails and protect your accounts at https://explore.plos.org/phishing.

If we can help with anything else, please email us at customercare@plos.org.

Thank you for submitting your work to PLOS ONE and supporting open access.

Kind regards,

PLOS ONE Editorial Office Staff

on behalf of

Professor Dr Hoh Boon-Peng

Academic Editor

PLOS One
